# Supplementary material for: Sporadic phage defense in epidemic Vibrio cholerae mediated by the toxin-antitoxin system DarTG is countered by a phage-encoded antitoxin mimic
Source: mBio. 2024 Sep 17;15(10):e00111-24. doi: 10.1128/mbio.00111-24 (PMC11481870; doi:10.1128/mbio.00111-24)
Supplement: Supplemental figures and tables — Figures S1-S11 and Tables S1-S4. [file mbio.00111-24-s0003.pdf]

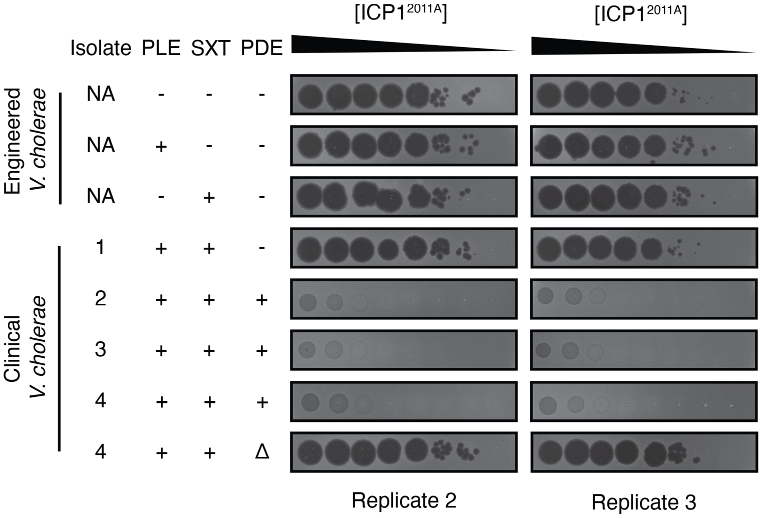

Figure S1: Biological replicates of ICP1^{2011A} spotted on *V. cholerae* harboring different phage defenses.

Ten-fold serial dilutions of ICP1^{2011A} were spotted on engineered or clinical *V. cholerae* strains containing PLE3, SXT *Vch*Ind6, or the PDE. Black zones of clearings are plaques. The opaque background is the *V. cholerae* lawn. Engineered strains are isogenic, while clinical isolates are not.

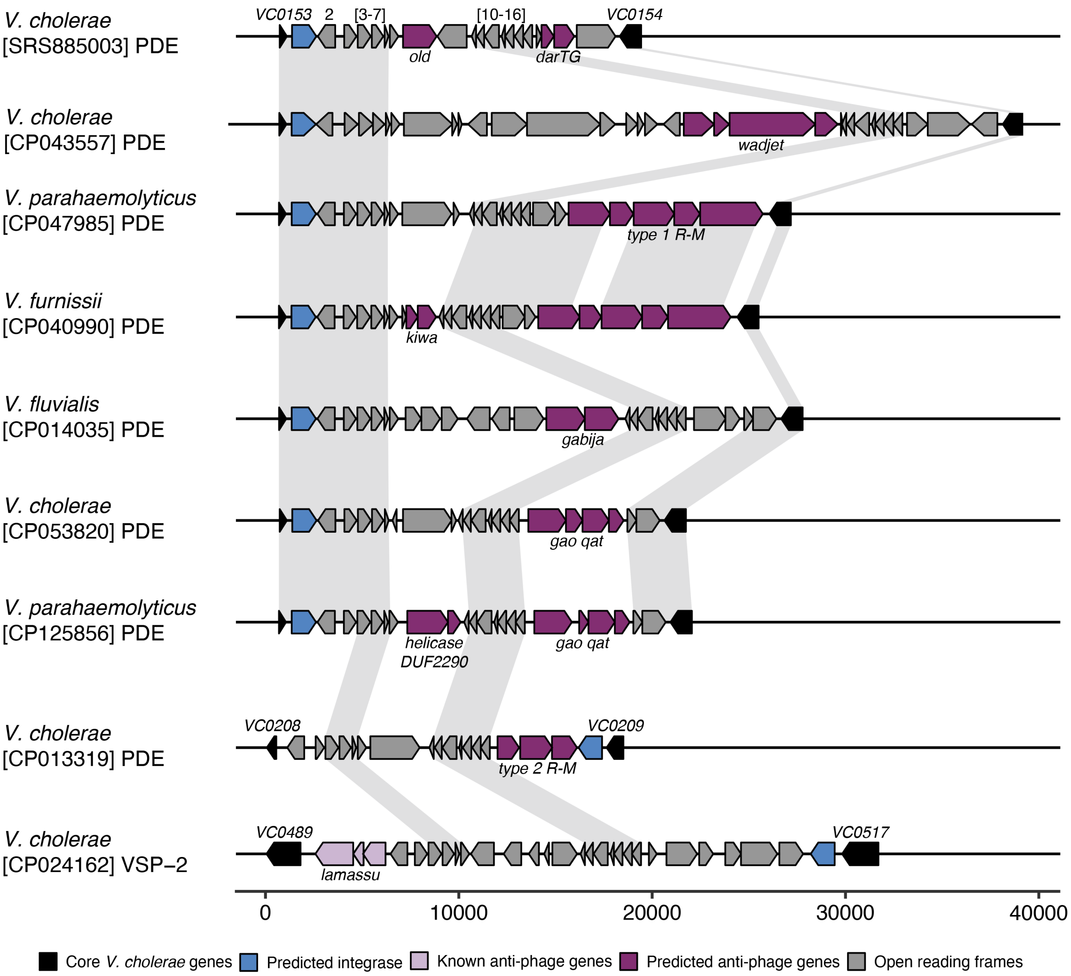

Figure S2: Elements related to the PDE also have putative anti-phage defense systems. Genetic maps (drawn to scale) of elements sharing nucleotide identify with the PDE characterized in this study (top). Defense Finder was used to predict if genes were involved in phage defense. Areas of >70% nucleotide identity are shaded in light grey. Accession numbers with the genomes containing these elements are written under the species name for each.

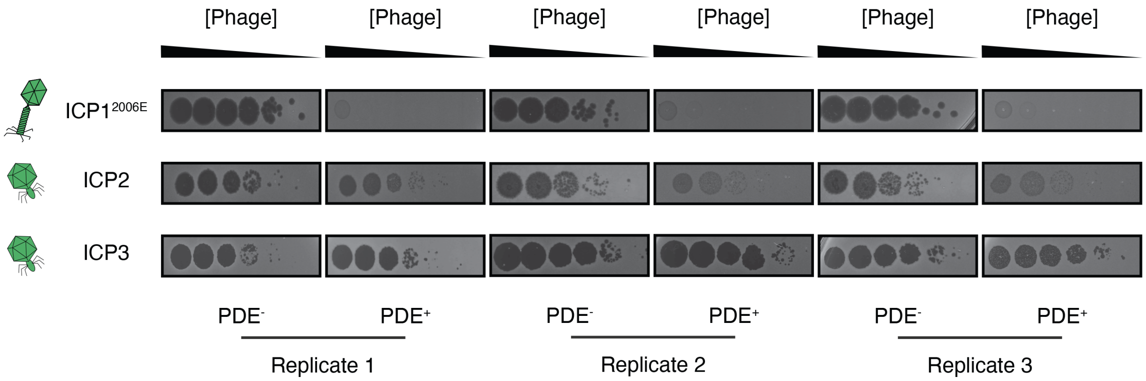

Figure S3: The PDE is sufficient to inhibit ICP1, but not all vibriophages. Ten-fold serial dilutions of ICP1^{2006E}, ICP2, and ICP3 were spotted on otherwise isogenic *V. cholerae* E7946 strains with and without the phage defense element (PDE). Black clearings are plaques where ICP1, ICP2, or ICP3 mounted a successful infection. The opaque background is the *V. cholerae* lawn.

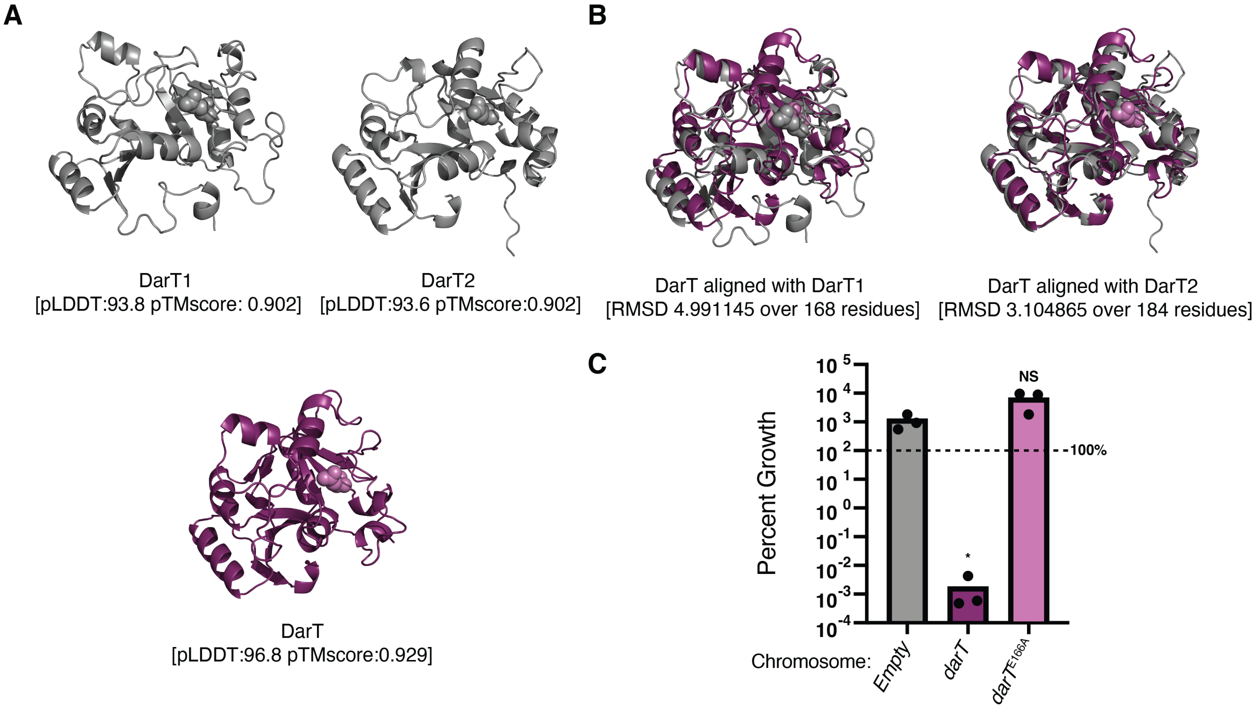

Figure S4: DarT ^{E166A} is not toxic to *V. cholerae.*

A) The ColabFold[39] predicted structures of DarT from *V. cholerae* (bottom, purple) and DarT1 and DarT2 from *E. coli* (grey). Conserved catalytic residues are shown as bubbles. pLDDT and pTM scores are written below the structures.

B) The superimposition of predicted structures of DarT from *V. cholerae* and the two DarTs from *E. coli*. The RMSD value is shown below the structure.

C) Percent growth as determined by colony-forming units of *V. cholerae* cells expressing the empty construct control, wild type DarT, or DarT^{E166A} via a chromosomal expression system for three hours. Each dot represents one biological replicate. The bar indicates the mean of all replicates. 100% cell survival is marked by the dotted line. *P = 0.0481 by ANOVA followed by Dunnett's T3 multiple comparisons test using the empty strain (not expressing anything) as the control condition, NS = nonsignificant.

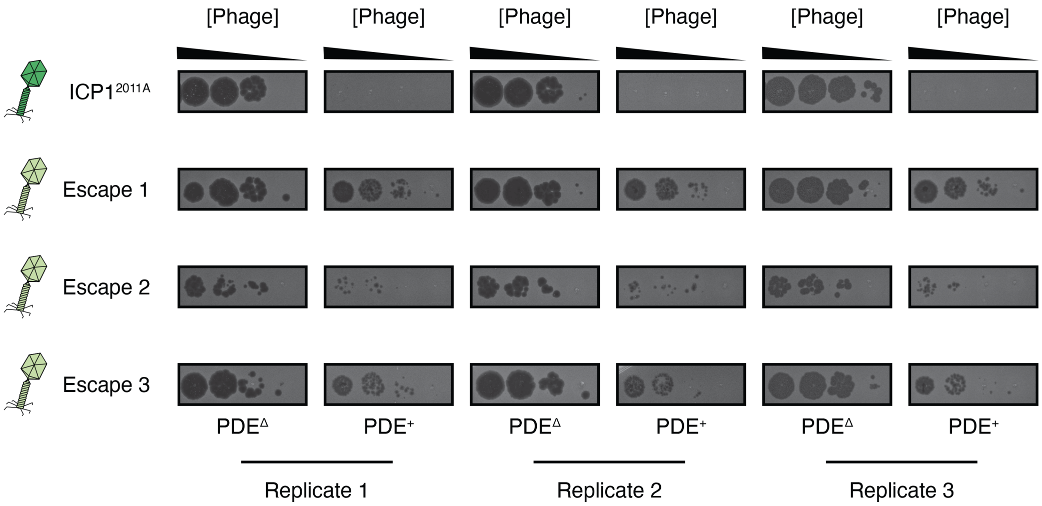

Figure S5: ICP1 escape mutants show improved plaquing on PDE+ clinical *V. cholerae.* Ten-fold serial dilutions of $ICP1^{2011A}$ and escape phage were spotted onto clinical *V. cholerae* strains encoding or lacking the phage defense element (PDE). Black clearings are where $ICP1^{2011A}$ was able to produce plaques. The opaque background is the *V. cholerae* lawn. Biological replicates are labeled.

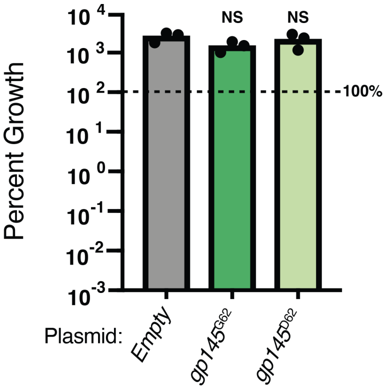

Figure S6: Neither allele of Gp145 induces DarTG-mediated cell death.
Percent growth as determined by colony-forming units of PDE$^+$ *V. cholerae* cells expressing an empty vector, Gp145^{G62}, or the evolved Gp145^{D62} variant for three hours. Each dot represents one biological replicate. The bar indicates the mean of all replicates. 100% cell survival is marked by the dotted line. ANOVA followed by Dunnett's T3 multiple comparisons test using the empty vector strain as the control condition shows that there are no statistically significant results, NS = nonsignificant.

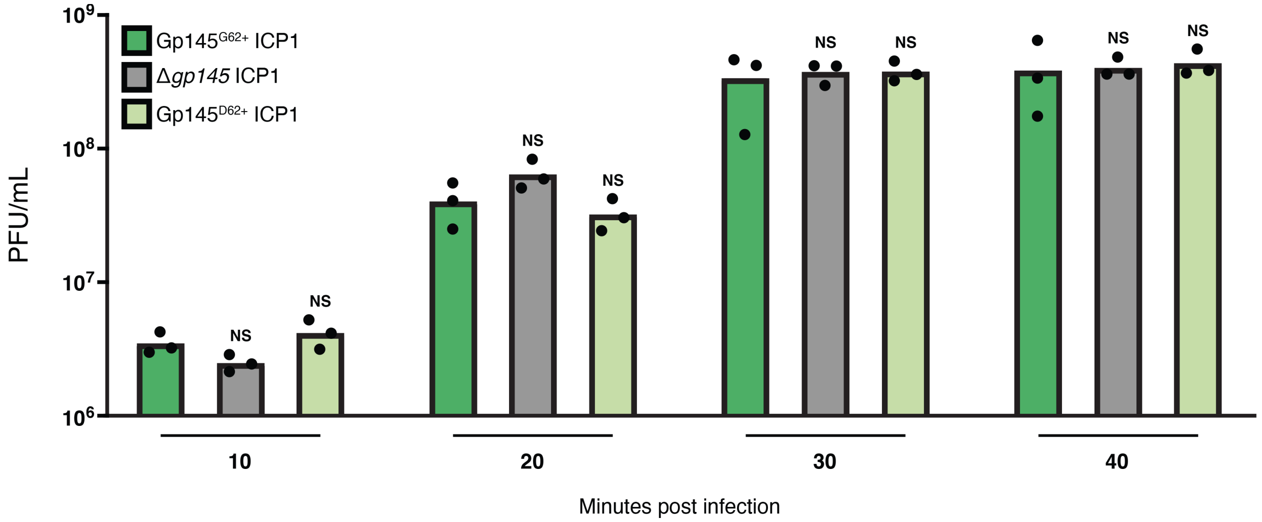

Figure S7: Engineered ICP1 with varying Gp145 alleles have no burst defect. One-step growth curve of phage ICP1^{2006E} (G145^{G62+}) and its mutant derivatives on PDE^{-} *V. cholerae*. Starting PFU values (~5E10^{6}) represent unabsorbed phage. Each dot represents one biological replicate, and the bar indicates the mean of all replicates. Two-way ANOVA followed by Dunnett's T3 multiple comparisons test using the Gp145^{G62+} ICP1^{2006E} infecting PDE^{-} *V. cholerae* as the control condition at each timepoint shows that there are no statistically significant differences, NS = nonsignificant.

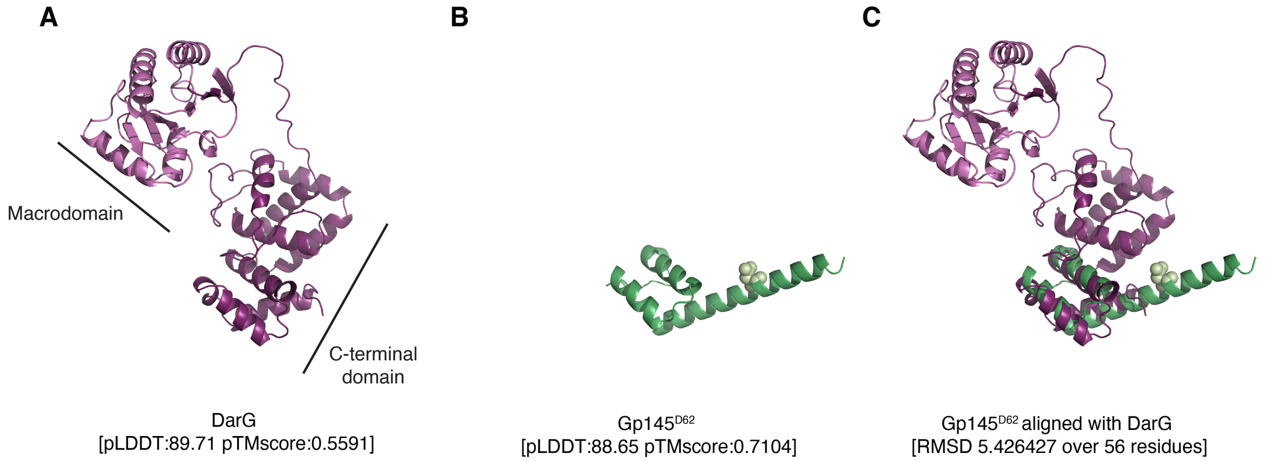

Figure S8: Gp145^{D62} and the C-terminus of DarG are predicted to be structurally similar.

A) The ColabFold[39] predicted structure of DarG reveals two major domains. The light purple domain (N-terminus domain) is the macrodomain, known for de-ADP-riboyslation. The dark purple domain is the C-terminus, which is known to bind to DarT in other systems[38]. pLDDT and pTM scores are written below the structure.

B) ColabFold predicted structure of Gp145^{D62} is shown in green. The D62 residue is shown in spheres and a lighter green color. pLDDT and pTM scores are written below the structure.

C) The superimposition of predicted structures of Gp145^{D62} and DarG reveals that the C-terminus of DarG and Gp145^{D62} show a striking structural similarity. The RMSD value is shown below the structure.

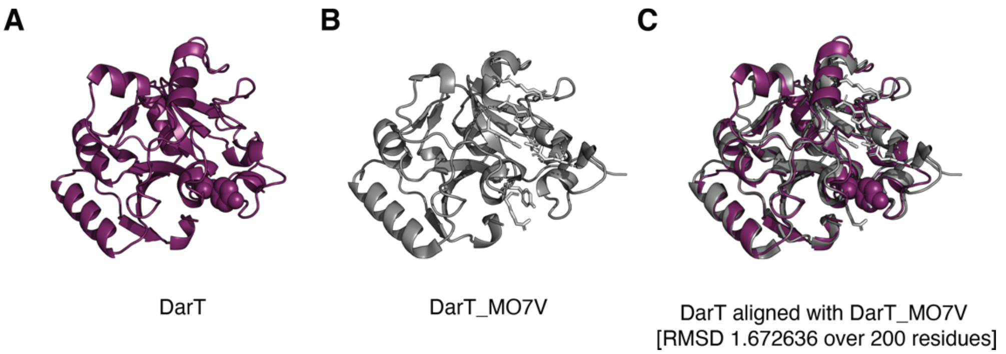

Figure S9: DarT^{Y82} falls in DarT's DNA binding site

A) The ColabFold[39] predicted structure of the DarT is shown in purple. The Y82 residue is shown in spheres.

B) The crystalized DarT (PDB: MO7V) is shown in grey. Residues involved in ssDNA binding are shown in lines in light grey.

C) The superimposition of predicted structures of DarT from *V. cholerae* and the crystalized DarT. The RMSD value is shown below the structure. DarT is colored in dark purple while the crystalized structure is colored in grey. The Y82 residue is shown in spheres and residues from DarT_MO7V involved in ssDNA binding are shown in lines in light grey. The Y82 residue from DarT aligns with the Y80 residue from DarT_MO7V.

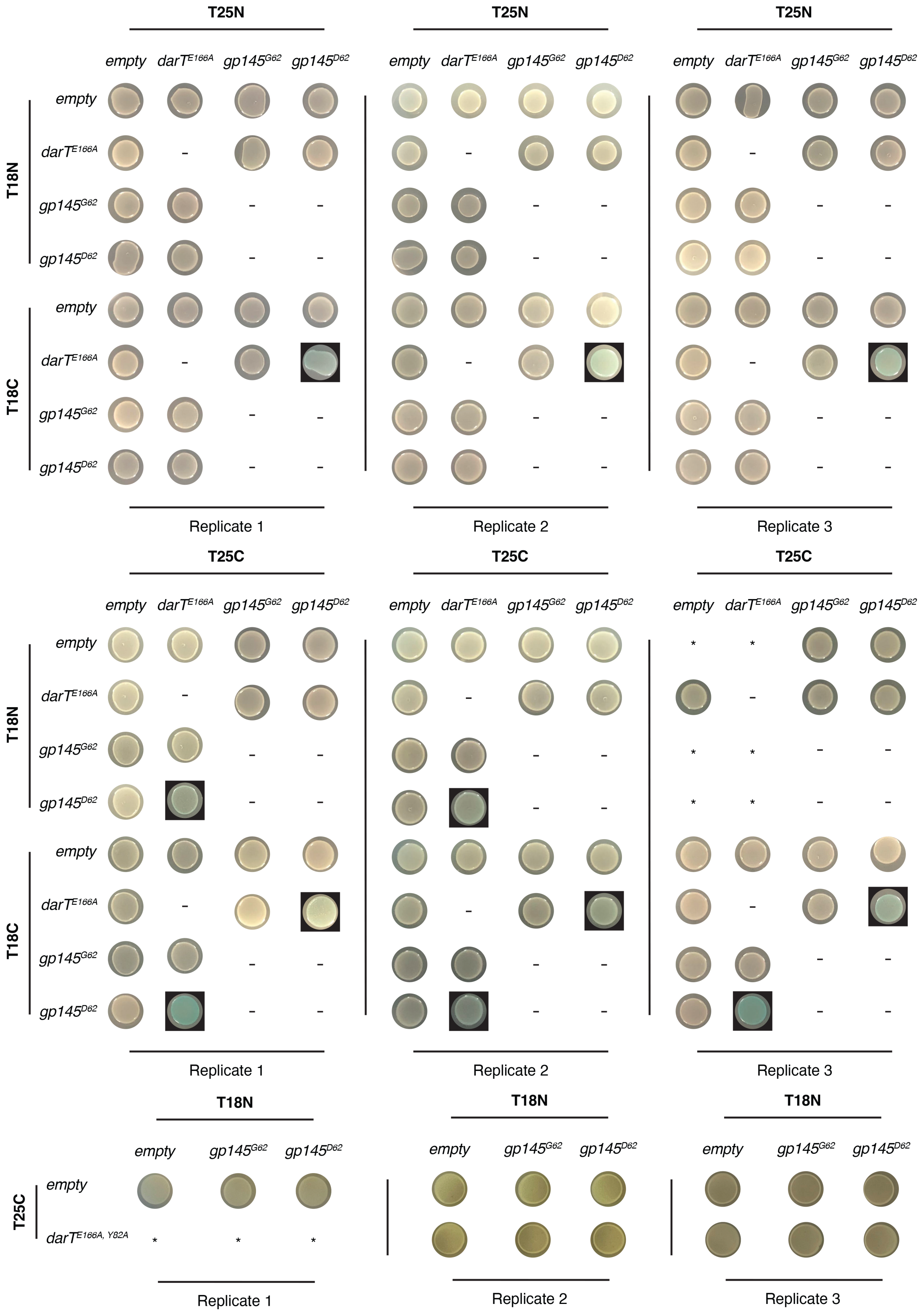
T25N
empty
darT^{E166A}
gp145^{G62}
gp145^{D62}
T18N
T18C
Replicate 1
Replicate 2
Replicate 3
T25C
T18N
T18C
Replicate 1
Replicate 2
Replicate 3
T18N
empty
gp145^{G62}
gp145^{D62}
T25C
darT$^{E166A, Y82A}$
Replicate 1
Replicate 2
Replicate 3

Figure S10: Replicates of bacterial two-hybrid data to detect protein-protein interactions between both alleles of Gp145, DarT^{E166A}, and $\text{DarT}^{E166A,\ Y82A}$.

Cells containing plasmids indicated on the top and left were spotted on agar plates containing X-gal and inducer. Blue spots indicate a physical interaction between the proteins fused to the CyaA subunits. Any blue colony is shown highlighted with a black box. Biological replicates are labeled. Any combination with a "-" were not tested, while the combinations with an "*" are shown in the main text.

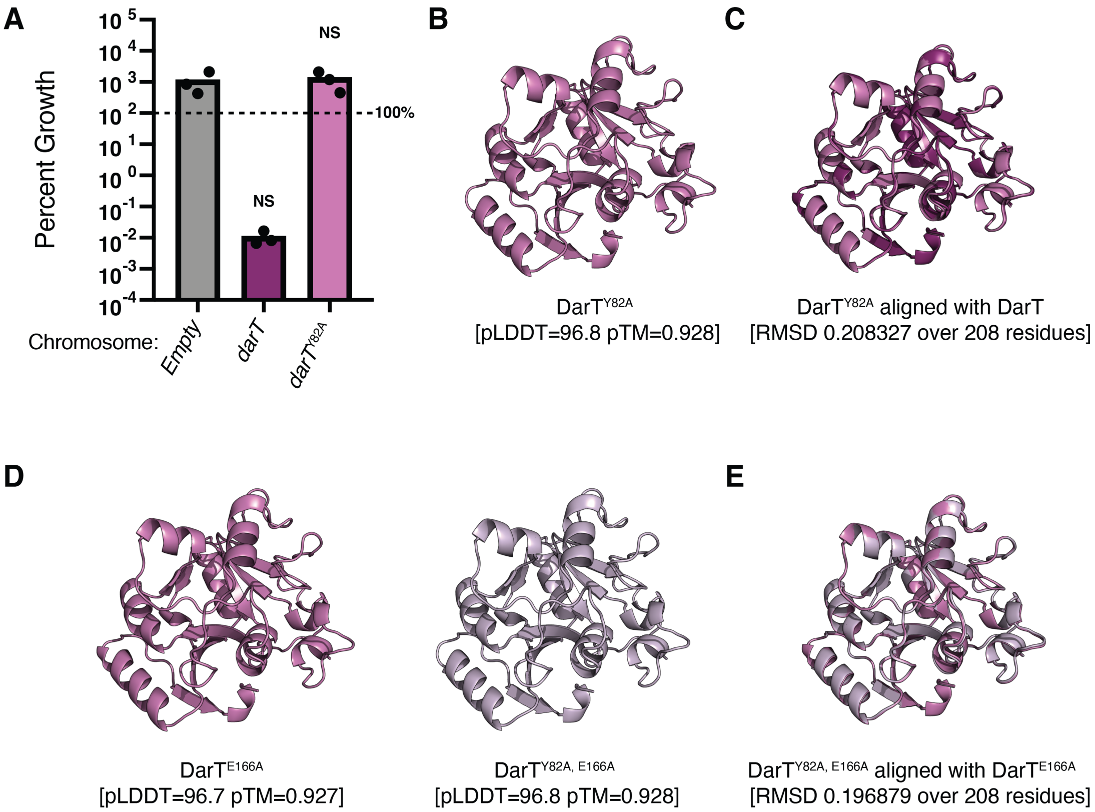

Figure S11: DarT^{Y82A} is not toxic *in vivo* but its predicted overall structure is retained compared to wild type.

A) Percent growth as determined by colony-forming units of *V. cholerae* cells expressing the empty construct control, wild type DarT, or DarT^{Y82A} via a chromosomal expression system for three hours. Each dot represents one biological replicate. The bar indicates the mean of all replicates. 100% cell survival is marked by the dotted line. NS = nonsignificant by ANOVA followed by Dunnett's T3 multiple comparisons test using the empty strain (not expressing anything) as the control condition.
B) The ColabFold[39] predicted structure of the DarT^{Y82A} mutant is shown in light purple.
C) The superimposition of predicted structures of wild type DarT from *V. cholerae* and the DarT^{Y82A} mutant. The RMSD value is shown below the structure. Wild type DarT is colored in dark purple while the mutant is colored in light purple.
D) The ColabFold[39] predicted structure of the DarT^{E166A} mutant is shown in light purple and the ColabFold[39] predicted structure of the DarT^{Y82A,E166A} double mutant is shown in muted purple. pLDDT and pTM scores are written below the structures.
E) The superimposition of predicted structures of the DarT^{E166A} mutant (light purple) and the DarT^{Y82A,E166A} double mutant (muted purple). The RMSD value is shown below the structure.

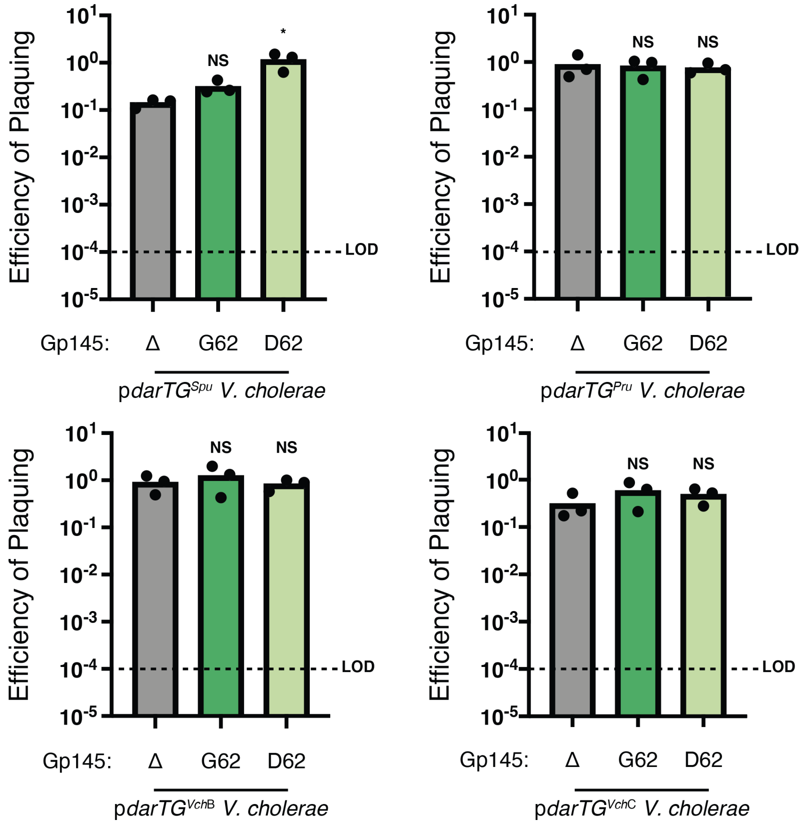

Figure S12: Diverse DarTG systems can but do not all inhibit ICP1 lacking Gp145 or expressing either $Gp145^{G62}$ or $Gp145^{D62}$.

The efficiency of plaquing (EOP) of engineered $ICP1^{2006E}$ with the allele of *gp145* indicated on *V. cholerae* expressing different DarTG systems from a plasmid (additional information for each DarTG system is in Supplemental Table 2). Each data point is representative of a single biological replicate, while the bar represents the mean of these replicates. LOD is the limit of detection. *P = 0.0075 by ANOVA followed by Dunnett's T3 multiple comparisons test using Δ*gp145* ICP1 as the control condition, NS = nonsignificant.

Supplemental Table 1: BreSeq analysis of ICP1^{2011A} escape mutants selected on PDE^{+} clinical *V. cholerae*.

**Position**	**Escape 1**[1]	**Escape 2**	**Escape 3**	**Annotation**	**Gene**[2]
14,612			6.00%	F72S (TTT→TCT)	Gp37 ←
17,642	100%			intergenic (-202/-36)	Gp46 ← / → Gp47
73,244	100%			E142G (GAG→GGG)	Gp124 ←
76,467			7.30%	F82Y (TTT→TAT)	Gp127 ←
76,470			7.10%	K81T (AAG→ACG)	Gp127 ←
76,473			7.00%	R80L (CGT→CTT)	Gp127 ←
76,476			7.40%	I79K (ATA→AAA)	Gp127 ←
80,258			5.60%	K56E (AAG→GAG)	Gp131 ←
**86,240**	**100%**	**100%**	**100%**	**G62D (GGT→GAT)**	**Gp145 ←**
112,450	100%			coding (1807-1851/1917 nt)	Gp203 →
[1]Frequency scores are the percentage of reads with the mutation compared to the total reads.					
[2]Arrows represent the direction of the strand. Arrows to the left indicate 3' to 5'. Arrows to the right indicate 5' to 3'.					

Supplemental Table 2: Summary of DarTG systems used in this study.

**Name**	**Species**	**DarT NCBI Reference Sequence**	**DarT Percent Identity to DarT**^{VchA}	**DarG NCBI Reference Sequence**	**DarG Percent Identity to DarG**^{VchA}
DarTG^{VchA}	*Vibrio cholerae*	See data sheet 1 for Amino Acid Sequence	100	See data sheet 1 for Amino Acid Sequence	100
DarTG^{Spu}	*Shewanella putrefaciens*	WP_011920083.1	80.84	WP_011920084.1	86.71
DarTG^{VchB}	*Vibrio cholerae*	WP_198302751.1	62.62	WP_096070761.1	79.83
DarTG^{PruA}	*Providencia rustigianni*	WP_006813742.1	65.89	WP_039854900.1	77.52
DarTG^{VchC}	*Vibrio cholerae*	WP_025795243.1	13.42	WP_057552482.1	24.31

Supplemental Table 3: Strains used in this study.
*Numbers refer to the reference listed in the main text. References with roman numerals in parenthesis are additional references listed below the tables.

Name in text	Strain	Description	Source*
Permissive PDE⁻ *V. cholerae*	KDS6	*Vibrio cholerae* E7946, El Tor Ogawa, O1, streptomycin resistant	Levine et al., 1982[(I)]
PLE3⁺ engineered *V. cholerae*	KDS38	*Vibrio cholerae* E7946 containing PLE3 integrated between VCA0415 and VCA0416.	O'Hara et al., 2017[24]
SXT ICE⁺ engineered *V. cholerae*	KL550	*Vibrio cholerae* E7946 containing SXT ICE *Vch*Ind6	LeGault et al., 2022[19]
Clinical *V. cholerae* isolate 1	KS511	*Vibrio cholerae* O1 clinical isolate from 2008 [SRR1944526]	Dalia et al., 2014[55]
Clinical *V. cholerae* isolate 2	KS515	*Vibrio cholerae* O1 clinical isolate from 2009 [SRR1944520]	Dalia et al., 2014[55]
Clinical *V. cholerae* isolate 3	KS516	*Vibrio cholerae* O1 clinical isolate from 2009 [SRR1944521]	Dalia et al., 2014[55]
Clinical *V. cholerae* isolate 4	KS39	*Vibrio cholerae* O1 clinical isolate from 2009 [SRR1944525]	Dalia et al., 2014[55]
Clinical *V. cholerae* isolate 4 ΔPDE	KMP1049	KS39 with an in-frame frt cassette replacing *orfs1-20* of the PDE	This study
Clinical *V. cholerae* isolate 4 Δ*darT*	KMP994	KS39 with an in-frame frt cassette replacing *darT* of the PDE	This study
PDE⁺ *V. cholerae*	KMP268	KDS6 containing the PDE integrated between VC0153 and VC0154	This study
PDE⁺ *V. cholerae* Δ*old*	KMP298	KMP268 with an in-frame frt cassette replacing *old*	This study
PDE⁺ *V. cholerae* Δ*darT*	KMP375	KMP268 with an in-frame frt cassette replacing *darT*	This study

PDE+ *V. cholerae* *darT*^{E166A}	KMP770	KMP268 (with an in-frame codon replacement (GAG->GCG) to have *DarT*^{E166A} expressed, *lacZ* replaced with Kanamycin resistance cassette integrated into *V. cholerae lacZ* locus	This study
PDE- *V. cholerae* chromosomal expression empty	KMP594	KDS6 with expression cassette (P_{BAD}-riboswitchE) from Dalia *et al*., 2020(II) integrated into *V. cholerae* lacZ locus with Kanamycin resistance cassette, expresses nothing (empty)	This study
PDE- *V. cholerae* chromosomal expression *darT*$^{VchA+}$	KMP596	KMP594 with DarT^{VchA} expressed from the expression cassette	This study
PDE- *V. cholerae* chromosomal expression *darT*$^{VchA\ E166A+}$	KMP592	KMP594 with DarT$^{VchA\ E166A}$ expressed from the expression cassette	This study
Empty Vector+ PDE- *V. cholerae* chromosomal expression *darT*$^{VchA+}$	KMP627	KMP596 containing an empty vector	This study
p*darG*$^{VchA+}$ PDE- *V. cholerae* chromosomal expression *darT*$^{VchA+}$	KMP630	KMP596 containing a vector that can express DarG^{VchA}	This study
p*gp145*$^{G62+}$ PDE- *V. cholerae* chromosomal expression *darT*$^{VchA+}$	KMP643	KMP596 containing a vector that can express Gp145^{G62}	This study
p*gp145*$^{D62+}$ PDE- *V. cholerae* chromosomal expression *darT*$^{VchA+}$	KMP631	KMP596 containing a vector that can express Gp145^{D62}	This study

PDE$^-$ *V. cholerae* chromosomal expression *darT*$^{Spu+}$	KMP1193	KMP594 with DarT^{Spu} expressed from the expression cassette	This study
Empty Vector$^+$ PDE$^-$ *V. cholerae* chromosomal expression *darT*$^{Spu+}$	KMP1195	KMP1193 containing an empty vector	This study
p*gp145*$^{G62+}$ PDE$^-$ *V. cholerae* chromosomal expression *darT*$^{Spu+}$	KMP1197	KMP1193 containing a vector that can express Gp145^{G62}	This study
p*gp145*$^{D62+}$ PDE$^-$ *V. cholerae* chromosomal expression *darT*$^{Spu+}$	KMP1199	KMP1195 containing a vector that can express Gp145^{D62}	This study
PDE$^-$ *V. cholerae* chromosomal expression *darT*$^{Pru+}$	KMP1526	KMP594 with DarT^{Pru} expressed from the expression cassette	This study
Empty Vector$^+$ PDE$^-$ *V. cholerae* chromosomal expression *darT*$^{Pru+}$	KMP1530	KMP1526 containing an empty vector	This study
p*gp145*$^{G62+}$ PDE$^-$ *V. cholerae* chromosomal expression *darT*$^{Pru+}$	KMP1532	KMP1526 containing a vector that can express Gp145^{G62}	This study
p*gp145*$^{D62+}$ PDE$^-$ *V. cholerae* chromosomal expression *darT*$^{Pru+}$	KMP1534	KMP1526 containing a vector that can express Gp145^{D62}	This study
PDE$^-$ *V. cholerae* chromosomal expression *darT*$^{Vch\mathrm{B}+}$	KMP1554	KMP594 with DarT$^{Vch\mathrm{B}}$ expressed from the expression cassette	This study

Empty Vector^{+} PDE^{-} *V. cholerae* chromosomal expression $darT^{Vch\mathrm{B}+}$	KMP1558	KMP1554 containing an empty vector	This study
p$gp145^{G62+}$ PDE^{-} *V. cholerae* chromosomal expression $darT^{Vch\mathrm{B}+}$	KMP1560	KMP1554 containing a vector that can express Gp145$^{\mathrm{G62}}$	This study
p$gp145^{D62+}$ PDE^{-} *V. cholerae* chromosomal expression $darT^{Vch\mathrm{B}+}$	KMP1562	KMP1554 containing a vector that can express Gp145$^{\mathrm{D62}}$	This study
PDE^{-} *V. cholerae* chromosomal expression $darT^{Vch\mathrm{C}+}$	KMP1556	KMP594 with DarT$^{Vch\mathrm{C}}$ expressed from the expression cassette	This study
Empty Vector^{+} PDE^{-} *V. cholerae* chromosomal expression $darT^{Vch\mathrm{C}+}$	KMP1564	KMP1556 containing an empty vector	This study
p$gp145^{G62+}$ PDE^{-} *V. cholerae* chromosomal expression $darT^{Vch\mathrm{C}+}$	KMP1566	KMP1556 containing a vector that can express Gp145$^{\mathrm{G62}}$	This study
p$gp145^{D62+}$ PDE^{-} *V. cholerae* chromosomal expression $darT^{Vch\mathrm{C}+}$	KMP1568	KMP1556 containing a vector that can express Gp145$^{\mathrm{D62}}$	This study
PDE^{-} *V. cholerae* chromosomal expression $darT^{Vch\mathrm{A\ Y82A}+}$	KMP1586	KMP594 with DarT$^{VchA\ \mathrm{Y82A}}$ expressed from the expression cassette	This study
BACTH host	KDS179	*E. coli* without adenylate cyclase gene used for BACTH	McKitterick and Seed, 2018[56]
ICP1$^{\mathrm{2011A}}$	KSϕ40	ICP1_2011_Dha_A WT Accession Number: MH310933.1	Seed et al., 2013[28]

ICP1^{2006E}	KSϕ36	ICP1_2006_Dha_E (Accession Number: MH310934.1) Δ*CRISPR* Δ*cas2-3*	McKitterick and Seed, 2018[56]
ICP2	ICP2	Accession Number: HQ641345	Seed et al., 2011[14]
ICP3	ICP3	Accession Number: HQ641340	Seed et al., 2011[14]
ICP1^{2006E} Δ*gp145*	KMPϕ34	KSΦ36 containing an in-frame deletion of *gp145*	This study
ICP1^{2006E} *gp145*^{D62}	KMPϕ38	KMPΦ34 where *gp145* locus has been repaired with *gp145*^{D62} and a 3X FLAG tag on the C-terminus	This study
ICP1^{2006E} *gp145*^{G62}	KMPϕ40	KMPΦ34 where *gp145* locus has been repaired g*p145*^{G62} a 3X FLAG tag on the C-terminus	This study
ICP1^{2011A} Escape 1	KMPϕ6	KSΦ36 that now possesses a mutation in Gp145, now codes for Gp145^{D62}, and possesses intergenic mutations and Gp124 mutation (described in Supplemental Table 1)	This study
ICP1^{2011A} Escape 2	KMPϕ9	KSΦ36 that now possesses a mutation in Gp145, now codes for Gp145^{D62}	This study
ICP1^{2011A} Escape 3	KMPϕ12	KSΦ36 that now possesses a mutation in Gp145, now codes for Gp145^{D62}	This study

Supplemental Table 4: Plasmids used in this study.

**Plasmids**	**Description and Identifier**	**Source**
Empty Vector	pMMB67EH engineered to contain an inducible riboswitch (E- induced by theophylline) downstream of a P_{tac} promoter, KS1864	Lab collection
p*darTG*$^{Vch\mathrm{A}}$	Empty vector engineered for the inducible expression of DarTG$^{Vch\mathrm{A}}$, KMP416	This study
p*darT*$^{\mathrm{E166A}}$*G*$^{Vch\mathrm{A}}$	Empty vector engineered for the inducible expression of DarT$^{\mathrm{E166A}}$G$^{Vch\mathrm{A}}$, KMP780	This study
p*gp145*^{G62}	Empty vector engineered for the inducible expression of Gp145$^{\mathrm{G62}}$, KMP418	This study
p*gp145*^{D62}	Empty vector engineered for the inducible expression of Gp145$^{\mathrm{D62}}$, KMP420	This study
p*darG*$^{Vch\mathrm{A}}$	Empty vector engineered for the inducible expression of DarG$^{Vch\mathrm{A}}$, KMP518	This study
empty pT18	pUT18, T18 subunit of *cya*	Lab collection
empty pT25	pKNT25, T25 subunit of *cya*	Lab collection
p*darT*$^{\mathrm{E166A}}$ - T25N	pKNT25, T25 subunit of *cya* fused to N-terminus of *darT*$^{\mathrm{E166A}}$, KMP701	This study
p*gp145*$^{\mathrm{G62}}$- T25N	pKNT25, T25 subunit of *cya* fused to N-terminus of *gp145*$^{\mathrm{G62}}$, KMP705	This study
p*gp145*$^{\mathrm{D62}}$- T25N	pKNT25, T25 subunit of *cya* fused to N-terminus of *gp145*$^{\mathrm{D62}}$, KMP707	This study
p*darT*$^{\mathrm{E166A}}$ - T18N	pUT18, T18 subunit of *cya* fused to N-terminus of *darT*$^{\mathrm{E166A}}$, KMP717	This study
p*gp145*$^{\mathrm{G62}}$- T18N	pUT18, T18 subunit of *cya* fused to N-terminus of *gp145*$^{\mathrm{G62}}$, KMP721	This study
p*gp145*$^{\mathrm{D62}}$- T18N	pUT18, T18 subunit of *cya* fused to N-terminus of *gp145*$^{\mathrm{D62}}$, KMP723	This study
p*darT*$^{\mathrm{E166A}}$ - T25C	pKNT25, T25 subunit of *cya* fused to C-terminus of *darT*$^{\mathrm{E166A}}$, KMP709	This study
p*darT*$^{\mathrm{E166A,\ Y82A}}$ - T25C	pKNT25, T25 subunit of *cya* fused to C-terminus of *darT*$^{\mathrm{E166A,\ Y82A}}$, KMP1626	This study
p*gp145*$^{\mathrm{G62}}$- T25C	pKNT25, T25 subunit of *cya* fused to C-terminus of *gp145*$^{\mathrm{G62}}$, KMP713	This study
p*gp145*$^{\mathrm{D62}}$- T25C	pKNT25, T25 subunit of *cya* fused to C-terminus of *gp145*$^{\mathrm{D62}}$, KMP715	This study

p*darT*^{E166A} - T18C	pUT18, T18 subunit of *cya* fused to C-terminus of *darT*^{E166A}, KMP725	This study
p*gp145*^{G62}-T18C	pUT18, T18 subunit of *cya* fused to C-terminus of *gp145*^{G62}, KMP729	This study
p*gp145*^{D62}-T18C	pUT18, T18 subunit of *cya* fused to C-terminus of *gp145*^{D62}, KMP731	This study
p*darTG*^{Spu}	Empty vector now engineered for the inducible expression of DarTG^{Spu} downstream of promoter and riboswitch, KMP699	This study
p*darG*^{Spu}	Empty vector now engineered for the inducible expression of DarG^{Spu} downstream of promoter and riboswitch, KMP749	This study
p*darTG*$^{Vch\mathrm{B}}$	Empty vector now engineered for the inducible expression of DarTG$^{Vch\mathrm{B}}$ downstream of promoter and riboswitch, KMP867	This study
p*darTG*^{Pru}	Empty vector now engineered for the inducible expression of DarTG^{Pru} downstream of promoter and riboswitch, KMP697	This study
p*darTG*$^{Vch\mathrm{C}}$	Empty vector now engineered for the inducible expression of DarTG$^{Vch\mathrm{A}}$ downstream of promoter and riboswitch, KMP855	This study

Additional references for supplementary tables:

(I) Levine, M. M. *et al.* The Pathogenicity of Nonenterotoxigenic Vibrio cholerae Serogroup 01 Biotype El Tor Isolated from Sewage Water in Brazil. *J. Infect. Dis.* **145**, 296–299 (1982).
